# Racial disparities in diabetes prevalence among cancer patients

**DOI:** 10.3389/fonc.2022.1099566

**Published:** 2023-01-13

**Authors:** Kimlin Tam Ashing, Gaole Song, Veronica Jones, Charles Brenner, Raynald Samoa

**Affiliations:** ^1^ Department of Population Sciences, Beckman Research Institute, City of Hope National Medical Center, Duarte, CA, United States; ^2^ Department of Surgery, City of Hope National Medical Center, California, CA, United States; ^3^ Department of Diabetes & Cancer Metabolism, City of Hope National Medical Center, California, CA, United States; ^4^ Department of Diabetes, Endocrinology & Metabolism, City of Hope National Medical Center, California, CA, United States

**Keywords:** racial disparities, health disparities, diabetes, cancer, healthcare

## Abstract

**Introduction:**

Cancer inequity is one of the most critical public health issues faced by ethnic minorities and people of lower socioeconomic status. The disparate burden of cancer is caused by poor access to care and inadequate delivery of cancer treatment, as well as comorbid and co-occurring conditions. Diabetes is a common and serious comorbid condition of cancer.

**Methods:**

To better understand diabetes prevalence among diverse cancer patients, this study analyzed and described characteristics of cancer patients with diabetes from local-level Service Planning Area (SPA) data using City of Hope Comprehensive Cancer Center data, and United States national-level data from The National Health Interview Survey.

**Results:**

Findings from national level data showed that patients in racial/ethnic minority groups had a higher occurrence of being diagnosed with diabetes, especially for non-Hispanic Blacks (OR=1.76, 95% CI=1.51, 2.03) and Hispanic/Latino individuals (OR=1.34, 95% CI=1.18, 1.52). Cancer patients who are older, ethnic minority, overweight/obese and with lower educational levels were more likely to have co-occurring diabetes. SPA-level patient data found similar results.

**Discussion:**

In response to our findings and other reports, clinicians and health system including health coverage organizations should routinely assess cancer patients for cooccurring chronic illnesses, in particular diabetes. Interventions improving coordinated care that integrates oncology, endocrinology and primary care, targeting cancer patients --especially racial/ethnic minorities, overweight/obese, and older patients who are at increased risk for diabetes -- ought to be considered as best practice Whole Person care. With coordinated care management, ethnic disparities in cancer may be better addressed and reduced. Additionally, policymakers can contribute by enacting policies improving access to and coverage of integrated oncology, chronic disease prevention, and associated specialty care i.e., endocrinology to equalize quality care for ethnic minority, lower educated, overweight/obese and older cancer patients who are more likely to suffer greater comorbidity, and inadequate oncology and coordinated care to reduce disparities.

## Introduction

Cancer is a critical public health issue. In the United States, it is estimated that 1.9 million new cancer cases will be diagnosed and 609,360 deaths will be caused by cancer in 2022 (1).. As the second leading cause of death in this country, the direct medical costs and indirect costs of cancer are huge. In 2015, the medical costs related to cancer were $183 billion and would increase to $246 billion by 2030 (2). For cancer patients, the burden is increased with co-occurring comorbid illnesses. Comorbidity can be defined as “the existence of a long-term health condition in the presence of a major disease of interest” (3). Evidence suggested the prevalence of comorbidities would increase with the years of survivorship increasing (4). The development of comorbidities is also relevant to the type of cancer: patients with lung cancer, kidney cancer, and stomach cancer are more likely to have other comorbid conditions (4). Comorbid conditions can complicate the treatment and outcome of cancer that negatively impact health-related quality of life and survival (4).

Diabetes is one of the most common and serious comorbid conditions. Cancer and diabetes, especially Type 2 diabetes, have many risk factors in common, including age, race/ethnicity, overweight/obesity, physical inactivity, smoking, etc. (5). These risk factors make patients with cancer more vulnerable to diabetes and the effects of diabetes. Additionally, some cancer treatments, such as certain targeted therapy treatments and chemotherapy drugs, can accelerate the development of diabetes or aggravate the process of diabetes (6). Diabetes becomes an important factor for patients and providers to choose the proper cancer treatment (6). Previous research indicated the mortality of cancer patients with diabetes was higher than cancer patients without any comorbid conditions (7). Social vulnerability index (SVI) characterizes non-medical factors that can impact a patient’s cancer outcome. In the cohort of patients who underwent stem cell transplantation, Hispanic and Asian patients showed an association with SVI and 1-year non-relapse mortality (NRM), while non-Hispanic whites showed no association with SVI and 1-year NRM. These findings highlight the important social-environmental factors play in health outcomes following hematopoietic cell transplantation (HCT), specifically among different racial and ethnic groups (8). For cancer patients with diabetes, more comprehensive healthcare services, including other medical specialties i.e., endocrinology, are necessary to meet patients’ special needs.

Since race/ethnicity is a major risk factor for both cancer and diabetes, some racial/ethnic groups have a higher risk of developing both diseases. For example, compared to other racial/ethnic groups, non-Hispanic Blacks have higher mortality for many types of cancer (9). Non-Hispanic Black and Hispanic/Latino women also have a higher prevalence of cervical cancer than women of other racial/ethnic groups (9). Meanwhile, the rates of diagnosed diabetes among American Indians/Alaskan Natives (14.5%), non-Hispanic Blacks (12.1%), and Hispanics (12.1%) are higher than other racial/ethnic groups (10). Previous research has indicated Latina breast cancer survivors had a higher risk of developing Type 2 Diabetes than the general population and diabetes was most prevalent among Latina survivors aged over 65 years old (11, 12).

Although high-quality healthcare is critical for all patients, many patients with cancer have various barriers to accessing healthcare services, especially racial/ethnic minority patients. The 2019 National Healthcare Quality and Disparities Report found that compared to non-Hispanic Whites, all other racial/ethnic groups reported receiving poorer healthcare quality (13). Even given comparable backgrounds such as income level, educational level, and insurance coverage status, racial/ethnic minority groups were reported to have disparities due to language barriers, provider bias, etc. (2). Therefore, more efforts are needed to improve the health of the minority patients with cancer, which is also an important component of improving the overall public health.

Because of the number of minority patients residing there, Los Angeles (LA) County serves as an ideal setting to develop and test measures to improve overall public health Based on 2020 U.S. Census Estimates, 48.32% of the total population of Los Angeles County was Hispanic or Latino, 14.83% was Asian, and 8.07% was Black (14). In addition, previous research highlighted racial/ethnic disparities in cancer prevalence and diabetes prevalence in Los Angeles County (15, 16). With a highly diverse population, it is important for Los Angeles County to ensure health equality among racial/ethnic minority populations and help them access high-quality healthcare. However, limited studies focused on the racial/ethnic disparity of the development of comorbid conditions among cancer patients. Cancer patients in racial/ethnic groups, especially those with comorbid conditions, should have a higher quality of care.

To have a better understanding of current diabetes prevalence among cancer patients, this study analyzed and described the diabetes prevalence among cancer patients from Service Planning Area (SPA) level and the national level. A SPA is a specific geographic area divided by the Department of Public Health of LA County to help provide better public health and clinical services tailored to the specific health needs of the residents in those different areas (17). Results of this study can provide insights into the outcomes of cancer patients who also had diabetes as a comorbid condition. It will address the severity of disparities in Los Angeles County. With the awareness of disparities in prevalence, policymakers can develop tailored policies to improve the quality of care for minority patients.

## Methods

### Data Source

This study is a secondary data analysis study. For national-level data, we used The National Health Interview Survey (NHIS), which is a cross-sectional household face-to-face interview survey program (18). NHIS collected health-related information among civilian noninstitutionalized populations residing within the 50 states and the District of Columbia in the United States (18). The most recent one-year NHIS data 2021 was used in this research (19). In 2021 NHIS, totally, 30,673 households had been interviewed including 29,482 sample adults and 8,261 sample children; the total household response rate was 52.8% (20).

For SPA-level data, we used patient data collected from the City of Hope Comprehensive Cancer Center (COH) from January 2020 to September 2022. COH is a private, not-for-profit clinical research center, hospital and graduate school to provide treatment and care services for patients with cancer, diabetes, and other life-threatening illnesses (21). Currently, COH’s cancer clinics cover more than 35 locations across Southern California, the United States, and provide care services for giving thousands of patients (22). Patient data were collected by clinicians and organized by the Research Informatics team. Due to a different design from the national-level survey program, patient records of COH only provided limited information. Thus, not all variables used from patient record data were the same as those from the national-level dataset.

### Measures

NHIS covers important health topics such as chronic diseases, health behaviors, health care status, use of preventive services, etc. The study population in this research is adults who have been diagnosed with cancer (any type). The dependent variable is the diagnosis of diabetes (any type) and the independent variable is self-reported race/ethnicity. Covariates included demographic characteristics (age, gender, marital status, educational attainment), healthcare coverage, BMI, and health behavior (tobacco use). NHIS questionnaire interviews the race and origin of participants and divides as *Hispanic/Latino, Non-Hispanic White only, Non-Hispanic Black/African American only, Non-Hispanic Asian only, Non-Hispanic American Indian and Alaska Native (AIAN) only, Non-Hispanic AIAN and any other group*, and *Other single and multiple races*. Though distinct groups that deserve focused attention, *Non-Hispanic AIAN only, Non-Hispanic AIAN and any other group, and Other single and multiple races* were merged into one category as *Other Single and Multiple Races* for statistical purposes only due to the small sample size. Cancer and diabetes diagnoses were self-reported on the survey questions: “Have you EVER been told by a doctor or other health professional that you had cancer?” and “Have you EVER been told by a doctor or other health professional that you had diabetes (Not including (gestational diabetes, prediabetes))” (23). Other covariate variables were recoded as age groups (18-34, 35-44, 45-54, 55-65, ≥65), marital status (married or live with a partner, other), educational attainment (did not graduate high school, graduated high school, attended college or technical school, and graduated from college or technical school), BMI (underweight, normal weight, overweight, and obese), tobacco use (current smoker, former smoker, and never smoker).

For SPA patient data, cancer diagnosis (any type) and diabetes diagnosis (any type) were reported by health professionals. Racial/ethnic information was self-reported and categorized as Hispanic/Latino, Non-Hispanic White only, Non-Hispanic Black/African American only, Non-Hispanic Asian only, and Other Single and Multiple Races. Covariate variables included in the patient record were age group (18-34, 35-44, 45-54, 55-65, ≥65), gender (female, male), healthcare insurance coverage (yes, no), BMI (overweight/obese, normal weight/underweight).

### Data analysis

For national-level analysis, since descriptive estimates in this research were obtained from a subpopulation (patients with cancer), descriptive analyses were conducted using methods for analyzing complex sample design data (24). Second-order (Satterthwaite) Rao-Scott chi-square tests were conducted to explore associations between the diagnosis of diabetes and race/ethnicity. Weighted multivariable logistic regression models were conducted using PROC SURVEYLOGISTIC to test associations between dependent and independent variables after controlling for covariates (24). All analyses were conducted using Statistical Analysis Software (SAS), version 9.4 (SAS Institute Inc., Cary, NC).

Since only limited information was included in patient records, for SAP-level analysis, descriptive analysis was conducted to show the percentage in different categories.

## Results

### National level

There were 3,654 adults diagnosed with cancer in the 2021 NHIS survey program. Among them, 603 also reported that they were diagnosed with diabetes. **Table 1A** presents demographic characteristics (age, gender, marital status, and educational attainment), healthcare coverage, and health behaviors (tobacco use, alcohol consumption, physical activity) among cancer patients with diabetes by racial and ethnic category. **Table 1B** presents characteristics among cancer patients without diabetes by racial and ethnic category. Based on the results, at the national level, more cancer patients with diabetes were aged over 65, especially patients in the non-Hispanic Asian group, with a percentage of 85%. Non -Hispanic Asian patients had higher educational levels than patients in other racial/ethnic groups, approximately 48% of them graduated from college or technical school. On the other hand, Hispanic/Latino patients had lower educational levels than others, about 33% of them did not graduate from high school; only 4.5% of them reported graduating from college or technical school. Over 80% of patients in all racial/ethnic groups had healthcare insurance coverage; however, Hispanic/Latino patients reported the lowest proportions of health insurance coverage which was 93.1%. Abnormal BMI was an important character for patients: more than half of all patients were overweight or obese; participatory, about 80% of patients in non-Hispanic White, non-Hispanic-Black, and Hispanic/Latino groups were overweight or obese.

**Table 1A:** Cancer Patients with/without Diabetes Characteristics by Racial and Ethnic Category, NHIS 2021^a,b^.

Characteristics	Non-Hispanic White N = 461 Weighted %	Non-Hispanic Black N = 67 Weighted %	Non-Hispanic Asian American N = 16 Weighted %	Hispanic/Latino N = 43 Weighted %	Other Single and Multiple Races N = 16 Weighted %
**Age**					
*45-54*	*7.7*	10.3	3.2	20.1	17.2
*55-64*	*19.1*	17.5	9.0	24.5	18.1
*≥ 65*	71.4	68.8	85.1	48.0	48.0
**Gender**					
*Male*	49.8	45.8	44.3	51.9	44.0
*Female*	50.2	54.2	55.7	48.1	56.9
**Marital Status**					
*Married or living with a partner*	63.2	38.5	77.8	50.4	38.7
*Other*	36.8	61.5	22.2	49.6	61.3
**Educational Attainment**					
*Did not graduate high school*	15.8	19.9	7.1	32.8	21.3
*Graduated high school*	28.6	25.3	33.4	26.0	18.3
*Attended college or technical school*	30.2	33.2	11.6	36.7	43.0
*Graduated from college or technical school*	25.5	21.7	47.8	4.5	17.5
**Healthcare Insurance Coverage**					
*Yes*	98.7	97.5	100.0	93.1	83.3
**BMI**					
*Overweight*	32.9	33.7	33.5	50.2	11.1
*Obese*	47.9	47.2	35.1	31.2	41.5
**Tobacco Use**					
***Current smoker***	**13.0**	**4.3**	**0**	**4.9**	**31.1**

^a^Numbers of all race/ethnicity do not add up to the total number of patients due to missing cases.

^b^Percentages may not add up equal to 100% due to rounding.

**Table 1B:** Cancer Patients without Diabetes Characteristics by Racial and Ethnic Category, NHIS 2021^a,b^.

Characteristics	Non-Hispanic White N = 2,638 Weighted %	Non-Hispanic Black N = 268 Weighted %	Non-Hispanic Asian American N = 53 Weighted %	Hispanic/Latino N = 137 Weighted %	Other Single and Multiple Races N = 53 Weighted %
**Age**					
45-54	10.4	7.9	21.9	16.1	10.2
55-64	21.2	19.4	13.2	20.7	7.6
≥ 65	58.6	57.4	48.8	36.9	54.1
**Gender**					
Male	44.4	43.3	20.4	42.2	33.1
Female	55.6	56.7	79.6	57.8	66.9
**Marital Status**					
Married or living with a partner	67.6	44.5	83.1	58.9	43.0
Other	32.4	55.5	16.9	41.1	57.0
**Educational Attainment**					
Did not graduate high school	7.0	14.1	7.1	25.9	7.3
Graduated high school	22.6	23.0	14.4	22.9	22.2
Attended college or technical school	26.0	33.4	19.3	31.6	40.7
Graduated from college or technical school	44.4	29.5	59.2	19.6	30.0
**Healthcare Insurance Coverage**					
Yes	97.7	96.8	100.0	93.0	95.1
**BMI**					
Overweight	35.4	36.7	20.8	42.9	21.8
Obese	27.9	38.5	6.5	30.3	33.2
**Tobacco Use**					
Current smoker	10.8	9.6	0	8.7	24.9

^a^Numbers of all race/ethnicity do not add up to the total number of patients due to missing cases.

^b^Percentages may not add up equal to 100% due to rounding.

**Table 2** shows the results of the multivariable logistic regression model assessing diabetes status as a co-occurring condition, which only included variables that were significantly associated with diabetes comorbidity. The overall Wald test suggested that all possible factors in the final model had statistically significant relations to diabetes comorbidity among cancer patients (F= 70.18, p<0.0001). Based on the results, age, race, gender, educational level, healthcare insurance coverage, BMI, and tobacco use were associated with diabetes comorbidity among cancer patients. Patients who are older, in minority groups, with lower educational levels would be more likely to be diagnosed with both cancer and diabetes at a statistical significance level. In addition, male patients were more likely to be diagnosed with cancer and diabetes (OR=1.25, 95% CI=1.11, 1.40). Cancer patients with abnormal BMI had significantly higher odds to co-occur with diabetes (overweight OR=2.01, 95% CI=1.70, 2.39; obese OR=3.89, 95% CI=3.31, 4.58). In terms of tobacco use, former smokers were more likely to develop diabetes (OR=1.20, 95% CI=1.06, 1.36). All ORs were adjusted.

**Table 2:** Results of Multivariable Analyses Predicting Prevalence of Diabetes among Cancer Patients by Racial and Ethnic Category, 2021 NHIS ^a^.

	Prevalence of Diabetes among Cancer Patients Unweighted N= 603			
	β	Adjusted OR	95% CI	*P*
Age (Years)				<.0001
**≥ 65 (Ref)**				
*45-54*	-0.87	0.42	(0.36, 0.50)	<.0001
*55-64*	-0.34	0.71	(0.63, 0.82)	
Race/ethnicity				<.0001
**Non-Hispanic White (Ref)**				
*Non-Hispanic Black*	0.62	1.86	(1.58, 2.18)	
*Non-Hispanic Asian American*	0.82	2.27	(1.79, 2.89)	
*Hispanic/Latino*	0.51	1.67	(1.43, 1.95)	
*Other*	0.74	2.09	(1.46, 2.98)	
Educational Attainment				<.0001
**Did not graduate high school (Ref)**				
*Graduated from college or technical school*	-0.80	0.45	(0.37, 0.54)	
*Attended college or technical school*	-0.32	0.72	(0.61, 0.86)	
*Graduated high school*	-0.22	0.80	(0.67, 0.95)	
Health Insurance Coverage				0.01
**No (Ref)**				
*Yes*	0.34	1.40	(1.10, 1.79)	
Gender				0.00
**Female (Ref)**				
*Male*	0.22	1.25	(1.11, 1.40)	
BMI				<.0001
**Normal weight (Ref)**				
*Overweight*	0.70	2.01	(1.70, 2.39)	
*Obese*	1.36	3.89	(3.31, 4.58)	
Tobacco Use				0.01
**Non-smoker (Ref)**				
*Current smoker*	-0.03	0.97	(0.82, 1.15)	
*Former smoker*	0.19	1.20	(1.06, 1.36)	

^a^Adjusted ORs were obtained after controlling for other predictor variables in the model.

**Table 3** presents the unadjusted odds ratio of diabetes diagnosis by racial/ethnic groups. Compared to non-Hispanic White cancer patients, patients in racial/ethnic minority groups had higher odds of being diagnosed with diabetes, especially for non-Hispanic Black patients (OR=1.76, 95% CI=1.51, 2.03) and Hispanic/Latino patients (OR=1.34, 95% CI=1.18, 1.52) with statistically significant higher odds.

**Table 3:** Diabetes Prevalence by Racial and Ethnic Category, 2021 NHIS^a,b^.

Characteristics	Non-Hispanic White N = 461 Unadjusted OR (95% CI)	Non-Hispanic Black N = 67 Unadjusted OR (95% CI)	Non-Hispanic Asian American N = 16 Unadjusted OR (95% CI)	Hispanic/Latino N = 34 Unadjusted OR (95% CI)	Other Single and Multiple Races N = 16 Unadjusted OR (95% CI)	P-value
Diabetes Diagnosis						<.0001
***Yes***	**Ref**	**1.76 (1.51, 2.03)**	**1.21 (0.99, 1.48)**	**1.34 (1.18, 1.52)**	**1.36 (0.97, 1.90)**	

^a^Race/ethnicity and diabetes diagnosis were included in the univariate model.

^b^ORs were unadjusted.

### SPA level

Totally, there were 41,692 patients with cancer from 2020 to 2022. Among them, 3,644 patients were also diagnosed with diabetes. **Table 4** presents the result of the descriptive analysis. Based on the results, the majority of cancer patients (86%) with diabetes were aged over 55 years old. There were more non-Hispanic White and Hispanic/Latino patients in the SPA dataset. In addition, most patients (87.7%) patients had healthcare insurance coverage. Also, more than 50% of them were overweight/obese.

**Table 4:** Cancer Patients with Diabetes Characteristics, Patient Data.

Characteristics	Cancer Patients with Diabetes N=3,644 Unweighted %	Cancer Patients with Diabetes Absolute Number
Age		
*18-34*	1.6	59
*35-44*	3.2	115
*45-54*	9.2	336
*55-64*	22.3	813
*≥ 65*	63.7	2,321
Gender		
*Male*	51.3	1,871
*Female*	48.7	1,773
Race/Ethnicity		
*Non-Hispanic White only*	37.2	1,355
*Non-Hispanic Black only*	5.8	211
*Non-Hispanic Asian only*	18.7	681
*Hispanic/Latino*	31.9	1,164
*Other*	6.4	233
Healthcare Insurance Coverage		
*Yes*	87.7	3,196
BMI		
***Overweight/Obese***	**65.0**	**2,370**

## Conclusion

Compared to non-Hispanic White cancer patients, cancer patients in minority groups have a higher rate of cooccurring diabetes. Based on national-level results, age, race, gender, educational level, healthcare insurance coverage, BMI, and tobacco use were associated with diabetes comorbidity. Particularly, cancer patients who are older, male, in racial/ethnic minority groups, with lower educational levels, and overweight/obese were more likely to develop diabetes as a comorbid condition. In addition, our SPA descriptive analysis results indicated similar status was found: more cancer patients with diabetes were older and overweight/obese.

## Discussion

Cancer and diabetes are both serious chronic diseases that lead to huge economic and societal burdens. If patients with cancer developed other comorbid conditions, their medical burden would be increased. Previous studies mainly focused on improving care quality for patients with cancer or diabetes solely, not for patients with both diseases. To have a better understanding of current diabetes prevalence among cancer patients, this study analyzed and described the diabetes prevalence among cancer patients from SPA level and national level.

Results of national-level analyses addressed the importance of various social determinants and provided suggestions for future health policies as well as interventions. This study found educational attainment is significantly associated with the development of diabetes among patients with cancers. One possible reason is patients with lower educational levels face barriers to preventive care. Abnormal BMI is another factor that is significantly associated with the development of diabetes. Healthcare providers and caregivers should address the importance of maintaining normal weight for patients with cancer. In addition, since our study found Hispanic/Latino patients had lower percentages of having healthcare insurance, it is a possible reason for the high prevalence of diabetes among Hispanic/Latino patients. Patient data from the COH cancer center found similar results with national-level analyses, which suggested consistency.

Based on our results, we strongly recommend future public health policies focus more on improving the quality of Whole Person Care. Whole Person Care is patient-centered and aims to improve health outcomes and well-being that cover physical, behavioral, emotional, and social services through the optimal use of different resources (25). Whole Person Care program also aims to improve care coordination services, develop healthcare delivery infrastructure, strengthen the collaboration among providers and communities, and share important data between various healthcare delivery organizations (26). For vulnerable patients, such as patients in minority racial/ethnic groups and patients with low socioeconomic status, Whole Person Care can take patients’ complex needs into account and provide comprehensive, coordinated care responsive to patients’ co-occurring illnesses.

Programming focused on MediCal-eligible community members who were homeless, justice-involved, or pregnant, and those with serious mental illnesses, substance use disorders, or complex health conditions.

This study has some limitations. First, patient data only contains limited information. Variables such as educational attainment, marital status, and health behaviors were not collected. Therefore, only descriptive analysis was conducted using patient data. Future research will conduct more statistical analyses when using more comprehensive patient data. Second, the BMI information in the NHIS dataset was self-reported, which may lead to recall bias. We considered using the National Health and Nutrition Examination Survey (NHANES) data since it includes both interviews and physical examinations and physiological measurements are collected by highly trained medical personnel, which will be more accurate (27). However, the most updated NHANES data is 2017-March 2020 Pre-pandemic cycle. Future studies can use new NHANES data when it releases for more accurate BMI information. Third, NHIS has a general question asking if participants had ever been diagnosed with diabetes (any type) and also a question asking if it is Type 1 or Type 2 diabetes. The majority of patients with diabetes were Type 2 diabetes. Considering the impact of analyses caused by sample size and missing responses, this study used the previous NHIS question and analyzed data from patients with diabetes of any type. Future studies will focus on Type 2 diabetes only.

Patients with cancer can have a co-occurring illness as well as develop other comorbid conditions, that increases their medical burden and impact their daily life. Diabetes is one of the most common and serious conditions, especially for patients who are ethnic/racial minority, older, overweight/obese and/or lower educated. In response to our findings and other reports, clinicians and health system including health coverage organizations should develop interventions improving Whole Person, coordinated care that integrates oncology and primary care, especially targeting cancer patients from racial/ethnic minority groups. Additionally, policymakers ought to enact policies improving access to and coverage of integrated primary, oncology, specialty care i.e., endocrine, to equalize quality care for vulnerable patients – who are more likely to suffer greater comorbidity, and inadequate oncology and coordinated care – to reduce disparities. Policies should also focus on facilitating chronic disease prevention programming such as the diabetes prevention programs aimed at engaging racial/ethnic minority (i.e., non-Hispanic Black and Hispanic/Latino), older, overweight/obese and lower educated patients. For cancer patients with diabetes or pre-diabetes symptoms, culturally informed and linguistically appropriate information and care should be provided.

## Data availability statement

Publicly available datasets were analyzed in this study. This data can be found here: The National Health Interview Survey (NHIS), publicly accessible https://www.cdc.gov/nchs/nhis/2021nhis.htm. Patient data (SPA level data) was collected by City of Hope National Medical Center and provided by disease informatics team.

## Ethics statement

The studies involving human participants were reviewed and approved by City of Hope Institutional Review Board. Written informed consent for participation was not required for this study in accordance with the national legislation and the institutional requirements.

## Author contributions

RS and VJ provided clinical expertise. GS, KA, and CB contributed to conceptualization and approach. GS conducted analysis. KA and GS worked on manuscript development. All authors overall review and editing. All authors contributed to the article and approved the submitted version.
